# Clinical Validation of a Deep Learning Algorithm for Detection of Pneumonia on Chest Radiographs in Emergency Department Patients with Acute Febrile Respiratory Illness

**DOI:** 10.3390/jcm9061981

**Published:** 2020-06-24

**Authors:** Jae Hyun Kim, Jin Young Kim, Gun Ha Kim, Donghoon Kang, In Jung Kim, Jeongkuk Seo, Jason R. Andrews, Chang Min Park

**Affiliations:** 1 Department of Radiology, Armed Forces Goyang Hospital, 215, Hyeeum-ro, Deogyang-gu, Goyang-si, Gyeonggi-do 10271, Korea; yyssaa21@gmail.com (J.H.K.); lwestsiderl@hanmail.net (J.Y.K.); idgunkim@gmail.com (G.H.K.); 2 Department of Internal Medicine, Armed Forces Goyang Hospital, 215, Hyeeum-ro, Deogyang-gu, Goyang-si, Gyeonggi-do 10271, Korea; etiria@catholic.ac.kr (D.K.); sginjung@naver.com (I.J.K.); jeong-gook@hanmail.net (J.S.); 3 Division of Infectious Diseases and Geographic Medicine, Stanford University School of Medicine, 291 Campus Drive, Stanford, CA 94305, USA; jasonandr@gmail.com; 4 Department of Radiology and Institute of Radiation Medicine, Seoul National University College of Medicine, 101 Daehak-ro, Jongno-gu, Seoul 03080, Korea

**Keywords:** acute febrile respiratory illness, emergency department, chest radiograph, artificial intelligence, deep learning algorithm

## Abstract

Early identification of pneumonia is essential in patients with acute febrile respiratory illness (FRI). We evaluated the performance and added value of a commercial deep learning (DL) algorithm in detecting pneumonia on chest radiographs (CRs) of patients visiting the emergency department (ED) with acute FRI. This single-centre, retrospective study included 377 consecutive patients who visited the ED and the resulting 387 CRs in August 2018–January 2019. The performance of a DL algorithm in detection of pneumonia on CRs was evaluated based on area under the receiver operating characteristics (AUROC) curves, sensitivity, specificity, negative predictive values (NPVs), and positive predictive values (PPVs). Three ED physicians independently reviewed CRs with observer performance test to detect pneumonia, which was re-evaluated with the algorithm eight weeks later. AUROC, sensitivity, and specificity measurements were compared between “DL algorithm” vs. “physicians-only” and between “physicians-only” vs. “physicians aided with the algorithm”. Among 377 patients, 83 (22.0%) had pneumonia. AUROC, sensitivity, specificity, PPV, and NPV of the algorithm for detection of pneumonia on CRs were 0.861, 58.3%, 94.4%, 74.2%, and 89.1%, respectively. For the detection of ‘visible pneumonia on CR’ (60 CRs from 59 patients), AUROC, sensitivity, specificity, PPV, and NPV were 0.940, 81.7%, 94.4%, 74.2%, and 96.3%, respectively. In the observer performance test, the algorithm performed better than the physicians for pneumonia (AUROC, 0.861 vs. 0.788, *p* = 0.017; specificity, 94.4% vs. 88.7%, *p* < 0.0001) and visible pneumonia (AUROC, 0.940 vs. 0.871, *p* = 0.007; sensitivity, 81.7% vs. 73.9%, *p* = 0.034; specificity, 94.4% vs. 88.7%, *p* < 0.0001). Detection of pneumonia (sensitivity, 82.2% vs. 53.2%, *p = 0*.008; specificity, 98.1% vs. 88.7%; *p* < 0.0001) and ‘visible pneumonia’ (sensitivity, 82.2% vs. 73.9%, *p* = 0.014; specificity, 98.1% vs. 88.7%, *p* < 0.0001) significantly improved when the algorithm was used by the physicians. Mean reading time for the physicians decreased from 165 to 101 min with the assistance of the algorithm. Thus, the DL algorithm showed a better diagnosis of pneumonia, particularly visible pneumonia on CR, and improved diagnosis by ED physicians in patients with acute FRI.

## 1. Introduction

Acute respiratory infections (ARIs) typically present as acute febrile respiratory illnesses (FRIs) cause approximately 4 million deaths worldwide each year [1]. In addition, ARIs were the second most common reason for emergency department (ED) visits in the United States in 2014 (18.2 per 1000 persons) [2], and chest radiographs (CR) have been the first-line imaging modality for diagnosing or excluding pneumonia [3]. It is very important to diagnose pneumonia in ARI patients because simple upper respiratory infections are usually self-limiting, while pneumonia can potentially lead to respiratory failure and intensive care unit admission without appropriate treatment [4]. However, it is challenging for the ED physicians to distinguish pneumonia from simple upper respiratory tract infections, mainly due to difficulties of CR interpretation. Several previous reports show substantial discrepancies in CR interpretation between the ED physicians and expert radiologists [5,6,7,8]. Unfortunately, it is not always possible to have full-time expert radiologists in every ED, especially on nights and weekends. Furthermore, CR interpretation in the ED should be timely for patient management [9], which is often challenging in reality.

Recently, deep-learning (DL) technology has been successfully applied in the medical field, particularly for the analysis of medical images [10] such as retinal photographs [11,12], pathology slides [13], and radiology images [14,15]. Hwang et al. developed and validated a DL algorithm for detection of major thoracic diseases including pneumonia on CRs [16], and it demonstrated excellent diagnostic performance with conveniently-collected datasets, surpassing expert radiologists. However, whether the DL algorithm can improve the CR interpretation of physicians in real-world clinical settings remains to be seen.

The purpose of our study was to evaluate the performance and added value of a commercially-available DL algorithm for detecting pneumonia on CRs from ED patients with acute FRI.

## 2. Materials and Methods

This retrospective study was approved by the ethics committee of the Armed Forces Medical Command (AFMC-18028-IRB-18-025), which waived the requirement for patients’ informed consent.

### 2.1. Patients and CR Collection

A total of 377 consecutive patients (375 men and 2 women, median age 20.0; interquartile range 20.0–21.0) with acute FRI (new or worsening episode of cough and fever of 38 °C or higher in the previous 24 h) underwent chest radiographs (387 CRs) in the ED of a tertiary military hospital in South Korea from August 2018 to January 2019 were studied. Among 377 acute FRI patients (387 CRs), 218 patients (222 CRs) were scanned by chest computed radiography (CT) within 24 h of the CRs. One author (J.H.K., with 6 years’ experience in CR interpretation) retrospectively reviewed all available medical records to select patients with acute FRI, and identify the available CRs and chest CT images of these patients.

All acute FRI patients in the present study underwent posteroanterior chest radiographs, acquired with a single dedicated radiography unit (GC85A, Samsung Healthcare, Seoul, Korea).

### 2.2. Laboratory Testing and Pathogen Detection

Bacterial culture was performed with the use of standard techniques on sputum samples. In addition, a real-time polymerase chain reaction (RT-PCR) assay was performed on throat swabs for the detection of adenovirus, influenza A and B viruses, human metapneumovirus (HMPV), parainfluenza virus types 1, 2, and 3, respiratory syncytial virus (RSV) A and B, human rhinovirus A, coronaviruses 229E, OC43, and NL63, human bocavirus 1/2/3/4, and human enterovirus. A bacterial pathogen was considered to be present if Gram-positive or Gram-negative bacteria were detected in the sputum sample in the culture. A viral pathogen was considered to be present if the RT-PCR assay for the virus tested positive.

### 2.3. DL Algorithm

We utilised a commercially available DL algorithm (Lunit INSIGHT for Chest Radiography, version 4.7.2; Lunit; accessible at https://insight.lunit.io). The algorithm was developed to detect major thoracic diseases including pulmonary malignancy, active pulmonary tuberculosis, pneumonia, and pneumothorax. It was developed with an image database consisting of 54,221 normal CRs and 35,613 CRs with major thoracic diseases (prevalence, 39.6%) [16]. The algorithm provided a probability score between 0 and 1 for the presence of the aforementioned thoracic diseases and created a heat map of the input CR to facilitate the localisation of the lesion. Among the two predefined cut-off values of the probability score (high-sensitivity and high-specificity cut-offs), we used a high-sensitivity cut-off (probability score of 0.16) for the binary classification of pneumonia in the present study. Although a high sensitivity could result in unnecessary antibiotic use, this decision was made considering that maintaining a high sensitivity is more important than high specificity in clinical practice, especially in the ED.

### 2.4. Reference Standards

The diagnosis of pneumonia in the present study was based on clinical, microbiological, and radiological information. Three radiologists (J.H.K., J.Y.K., and K.H.K., each with 5–8 years’ experience in CR interpretation) independently determined whether patients had radiological evidence of pneumonia or not by retrospective review of CRs, and/or CT imaging along with any available clinical information and laboratory tests.

In addition, patients were classified as having “visible pneumonia on CR” if radiologists identified consolidation or other infiltration (linear or patchy alveolar or interstitial densities) on CR. Therefore, patients with evidence of pneumonia on CT scans but not CRs were excluded from “visible pneumonia on CR”. In case of discordant interpretation among the three radiologists, they re-evaluated the CRs and/or CTs, and came to a consensus.

Evaluation of the lesion localisation accuracy was done by a board-certified radiologist (J.H.K.), who reviewed all heat map images and determined if the DL algorithm was correct. Classifications made by the DL algorithm were only considered correct when the lesion locations were accurate.

### 2.5. Observer Performance Assessment

ED CRs were routinely read by physicians (board-certified internists) in our hospital; therefore, we decided to conduct an observer performance test for ED physicians to simulate clinical practice. The performance assessment included 2 sessions, and in both, the observers read the CRs in the radiologist’s reading room with a high-resolution radiology monitor (MS53i2; Totoku, Tokyo, Japan) without any time limit. In session 1, three ED physicians with 6–7 years of experience in interpretation of ED CRs were asked to independently grade all the CRs on a 5-point scale for the presence of pneumonia, as follows: 1 = definitely normal, 2 = probably normal, 3 = indeterminate, 4 = probably pneumonia, and 5 = definitely pneumonia. The physicians were aware that each patient had acute FRI, and that the CRs were acquired for that purpose. Eight weeks after session 1, the three physicians independently reassessed every CR with the assistance of the DL algorithm to assign a grade (according to the 5-point scale) corresponding to the presence of pneumonia (second session). The probability scores of pneumonia and heat map images of the DL algorithm were provided on each CR interpretation in session 2.

The total observer reading time at each session was recorded.

### 2.6. Statistical Analysis

We calculated diagnostic performances of the DL algorithm and the physicians in terms of the following two tasks: (a) Detection of pneumonia on CRs irrespective of its visibility on CRs, (b) Detection of visible pneumonia on CR.

Receiver operating characteristic curves were constructed and area under the receiver operating characteristics curves (AUROCs) was calculated with 95% confidence intervals (CIs) by using the method of DeLong et al. [17]. The sensitivity, specificity, positive predictive values (PPVs), and negative predictive values (NPVs) of the DL algorithm were calculated according to the high-sensitivity cut-off value (probability score of 0·16). Observer interpretation with scores ≥3 were regarded as positive. A threshold of score ≥3 was chosen through maximization of the F1 score on the pooled data of three observers from session 1. The McNemar test was used to compare the sensitivity and specificity of the different methods.

To evaluate clinical characteristics data, distribution normality was assessed using the Kolmogorov-Smirnov test. Non-normally distributed data were presented as median (interquartile range) and categorical variables as frequency (%). Differences between pneumonia and non-pneumonia groups were analyzed by Fisher’s exact test (for categorical data) or Mann–Whitney U test (for continuous data, but not normally distributed).

Statistical analyses were performed with a software (MedCalc, version 19.0.3; MedCalc Software, Mariakerke, Belgium). *p* values were two-sided, and *p* < 0.05 indicated a statistically significant difference.

## 3. Results

The clinical characteristics of acute FRI patients are summarized in Table 1.

**Figure 1:**
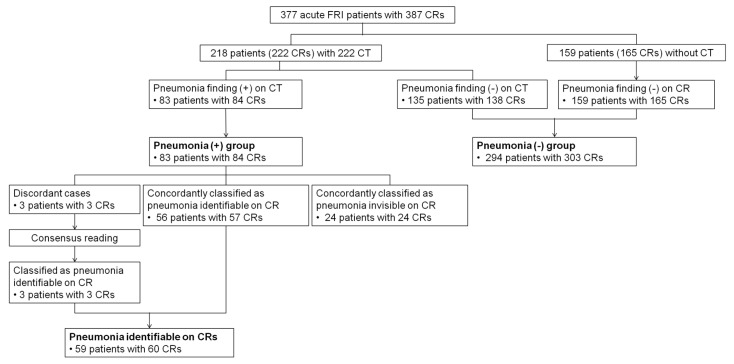
Flow chart for the determination of reference standard. FRI = febrile respiratory illness, CR = chest radiograph, CT = computed tomography.

Among 377 acute FRI patients (387 CRs), 83 patients with 84 CRs were diagnosed with pneumonia, which were confirmed with chest CT scans (Figure 1). Among 83 pneumonia patients, 59 patients (60 CRs) were designated as “visible pneumonia on CR”, and the remaining 24 patients (24 CRs) were designated as “invisible pneumonia on CR” (Figure 1).

Fifty-eight out of the 83 pneumonia patients underwent tests for causative pathogens and one or more viruses were detected in 31 patients (53%): adenovirus (*n* = 19); human rhinovirus (*n* = 4); human enterovirus (*n* = 2); influenza A virus (*n* = 1); coronavirus NL63 (*n* = 1); parainfluenza virus type 2 (*n* = 1); adenovirus and coronavirus OC43 (*n* = 2); adenovirus and human rhinovirus (*n* = 1).

### 3.1. Pneumonia Detection Performance of the Deep-Learning Algorithm on CRs

**Figure 2:**
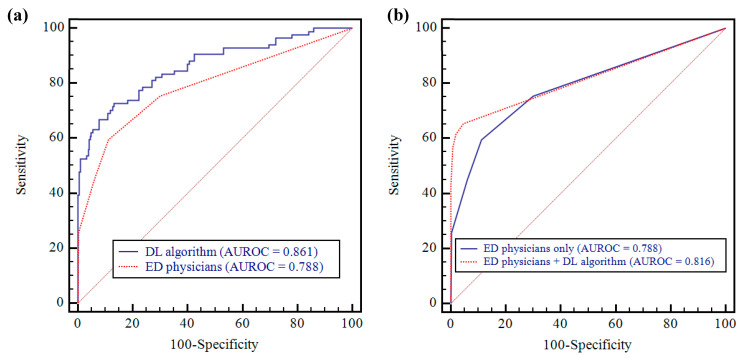
AUROCs of DL algorithm and ED physicians (pneumonia vs. non-pneumonia). (**a**) The DL algorithm showed significantly higher performance than that for ED physicians (0.861 vs. 0.788; *p* = 0.019). (**b**) ED physicians’ performance was improved after assistance with DL algorithm (0.788 vs. 0.816; *p* = 0.068). AUROC = area under the receiver operating characteristics curve, DL = deep learning, ED = emergency department.

AUROC of the algorithm for pneumonia detection was 0.861 (95% CI: 0.823–0.894) (Figure 2) (Table 2). The algorithm’s sensitivity, specificity, PPV, and NPV were 58.3% (95% CI: 47.1–69.0%), 94.4% (95% CI: 91.2–96.7%), 74.2% (95% CI: 63.7–82.6%), and 89.1% (95% CI: 86.4–91.3%), respectively.

**Figure 3:**
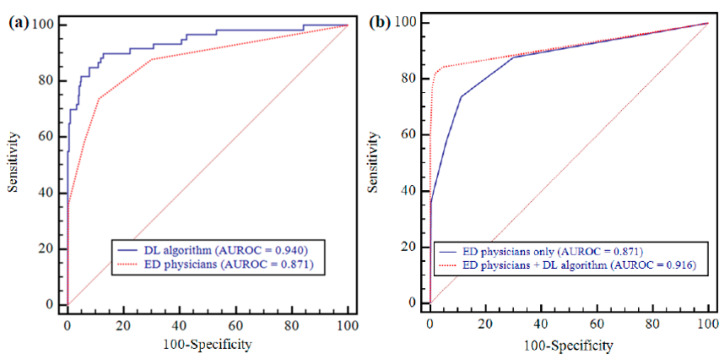
AUROCs of DL algorithm and ED physicians (visible pneumonia on CR vs. non-pneumonia). (**a**) The DL algorithm showed significantly higher performance than that for ED physicians (0.940 vs. 0.871; *p* = 0.007). (**b**) ED physicians’ performance was significantly improved after assistance with DL algorithm (0.871 vs. 0.916; *p* = 0.002).

As for the detection of ‘visible pneumonia on CR’, AUROC of the algorithm was 0.940 (95% CI: 0.910–0.962) (Figure 3) (Table 3). The algorithm’s sensitivity, specificity, PPV, and NPV were 81.7% (95% CI: 69.6–90.5%), 94.4% (95% CI: 91.2–96.7%), 74.2% (95% CI: 64.1–82.3%), and 96.3% (95% CI: 93.8–97.8%), respectively.

### 3.2. Performance Comparison between Deep-Learning Algorithm and Physicians

There was a statistically significant difference between AUROC of the DL algorithm and the pooled AUROC from the three observers for the detection of pneumonia (0.861 vs. 0.788 [95% CI: 0.763–0.811]; *p* = 0.017) (Figure 2) (Table 2). The specificity of the algorithm was significantly higher than that of the observers (94.4% vs. 88.7%; *p* < 0.0001), and the algorithm’s sensitivity was also greater than that of the observers but did not achieve statistical significance (58.3% vs. 53.2%; *p* = 0.053) (Table 2).

As for the detection of ‘visible pneumonia on CR’, the algorithm’s AUROC was significantly higher than that of the three physicians (0.940 vs. 0.871; 95% CI: 0.849–0.890; *p* = 0.007) (Figure 3) (Table 3). The sensitivity and specificity of DL algorithm were significantly higher than those of the observers (81.7% vs. 73.9%, and 94.4% vs. 88.7%; *p* = 0.034 and < 0.0001, respectively) (Table 3).

Diagnostic performances of the algorithm and individual physician are summarized in Table 2 and Table 3.

### 3.3. Performance Comparison between Physicians-only and Physicians Aided by the Algorithm

With regard to the detection of pneumonia, the performance of physicians assisted by the algorithm was higher than those of physicians-only (AUROC; 0.816 [95% CI: 0.793–0.838] vs. 0.788), but the difference was not statistically significant (*p* = 0.068) (Figure 2) (Table 2). The pooled sensitivity and specificity of physicians assisted by the algorithm were significantly higher than those of physicians-only (0.599; 95% CI: 0.536–0.660 vs. 0.532, and 0.981; 95% CI: 0.970–0.989 vs. 0.887; *p* = 0.008 and < 0.0001, respectively).

Mean total reading time of the physicians with the assistance of the algorithm was reduced by 64 min from 165 to 101 min (Table 2).

**Figure 4:**
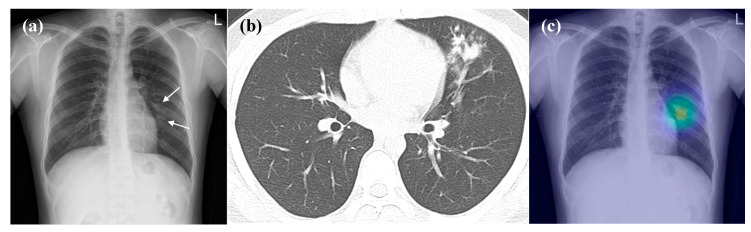
Representative case of the observer performance test. (**a**) The CR demonstrates patchy opacity in the left middle lung field (arrow), which was initially detected by only one of three observers. (**b**) The CT taken on the same day shows branching opacities and centrilobular nodules at the left upper lobe. (**c**) The DL algorithm correctly detected the lesion (probability score, 0.577). After assistance from the DL algorithm, all observers detected the lesion.

As for detection of ‘visible pneumonia on CR’, the pooled AUROC from the physicians significantly improved with the assistance of the algorithm (0.916 [95% CI: 0.898–0.931] vs. 0.871, *p* = 0.002) (Figure 3 and Figure 4) (Table 3). The pooled sensitivity and specificity from physicians assisted by the algorithm were also significantly enhanced compared with those of physicians-only (82.2% [95% CI: 75.8–87.5%] vs. 73.9%, and 98.1% [95% CI: 97.0–98.9%] vs. 88.7%; *p* = 0.014 and < 0.0001, respectively).

### 3.4. False-Positive Interpretations of DL Algorithm (Detection of Pneumonia on CRs)

**Figure 5:**
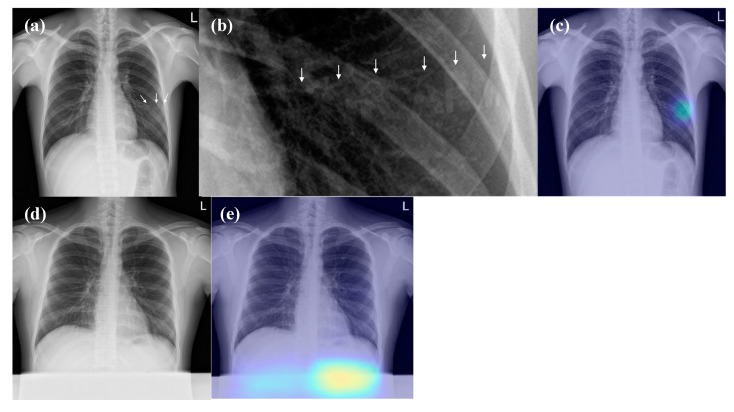
False positive interpretations of the DL algorithm. (**a**,**b**) The CR shows radio-opaque letters “ROK ARMY” (arrows) of the shirt at the left middle lung field. (**c**) The DL algorithm wrongly localised the radio-opaque letters (probability score, 0.348). (**d**) There is an accidentally included abdominal shield at the lower part of the CR. (**e**) The DL algorithm wrongly detected the abdominal shield (probability score, 0.684). None of the three observers identified these foreign bodies as lesions.

There were 21 false-positive results of the DL algorithm, which are detailed below: normal vascular marking (*n* = 6); normal lung apex (*n* = 2); normal costophrenic angle (*n* = 1); bone island (*n* = 4); calcified nodule (*n* = 1); radio-opaque letters on a shirt (*n* = 6) (Figure 5); abdominal shield (*n* = 1) (Figure 5).

## 4. Discussion

In the present study, the DL algorithm demonstrated fair diagnostic performance in detecting pneumonia (AUROC, 0.861) by evaluating CRs in a consecutive patient acute FRI cohort. However, the sensitivity of the DL algorithm was only 58.3%; a result that can be sufficiently explained given that 24 of the 83 pneumonia patients had a form of pneumonia that was not visible on the concurrent CRs. With respect to detecting ‘visible pneumonia on CR’, the DL algorithm demonstrated excellent diagnostic performance (AUROC, 0.936). It is comparable to the diagnostic performance of thoracic radiologists to detect major thoracic diseases in a previous study (AUROC, 0.932) [16] and the diagnostic performance of the DL algorithm (AUROC, 0.95) for detection of clinically relevant abnormalities in the ED of a general tertiary hospital [9]. In addition, our results showed that the algorithm significantly improved the diagnostic performance of ED physicians in the detection of pneumonia on CRs. Additionally, for detecting pneumonia, pooled sensitivity and specificity of the ED physicians significantly improved with the assistance of the algorithm. In the detection of ‘visible pneumonia on CR’, pooled AUROC, sensitivity, and specificity of the ED physicians were significantly enhanced by the algorithm’s assistance. These results are similar to the previous studies [9,16].

Interestingly, the DL algorithm not only improved the diagnostic performance, but also substantially reduced the reading time of CR interpretation by the ED physicians (mean total reading time, 165 min vs. 101 min). Consequently, with the assistance of the DL algorithm ED physicians could detect pneumonia on CRs more quickly and accurately. Furthermore, if the DL algorithm provisionally analyzed ED CRs and if there is an alerting system for clinically-critical or relevant diseases, ED physicians could prioritize CRs with a high-probability score of clinically relevant abnormalities (such as pneumonia). This would shorten the turnaround time from acquisition to interpretation and enable timely treatment of these patients. Therefore, we believe that the DL algorithm in the present study could improve the quality of pneumonia care in patients with acute FRI such as COVID-19 [18] by improving the diagnostic accuracy and reducing the time to diagnosis.

Regarding the diagnostic performance boosting effect of the algorithm, there was variability across the three ED physicians. There was a significant improvement in the specificity of detection of pneumonia and ‘visible pneumonia on CRs’ in observers 1 and 2, but there was no significant improvement in the specificity of observer 3 with the assistance of DL algorithm. In addition, only observer 1 showed a significant improvement of AUROC and observer 3 alone showed a significant improvement in sensitivity with the assistance of the DL algorithm for the detection of ‘visible pneumonia on CR’. Although the diagnostic performance of ED physicians generally improved and the reading time decreased after using the DL algorithm, the variability in the effectiveness of assistance across individual physicians should be considered when using it in clinical practice.

It is noteworthy that the DL algorithm showed several unexpected false-positives. Specifically, the algorithm misinterpreted the radio-opaque letters on a shirt (*n* = 6) and abdominal shield (*n* = 1) as abnormal lesions, which would have been easily ignored by physicians; none of the observers considered those foreign materials as abnormal lesions. Physicians should be aware of this problem when utilising the DL algorithm in their clinical practice and the developers should correct this shortcoming.

The present study had several limitations. Firstly, the majority of our study cohort consisted of young men without underlying disease (370 men and 2 women, median age 20 [interquartile range 20.0–21.0) and moreover military hospital patients constitute a specialised population. Further investigations are needed to validate the diagnostic performance of the DL algorithm in acute FRI patients from the general population. Secondly, we performed the observer performance assessment on three physicians. In addition, since there was an inter-observer variability regarding the effect of DL algorithm assistance, further performance tests on multiple observers are needed to validate the results of the present study.

In conclusion, the DL algorithm showed fair diagnostic performance for detecting pneumonia, particularly visible pneumonia on CR, and improved the diagnostic performance of ED physicians in patients with acute FRI.

## Figures and Tables

**Table 1:** Clinical Characteristics of Patients with Acute Febrile Respiratory Illness.

		All Patients (*n* = 377)	Pneumonia (*n* = 83)	Non-Pneumonia (*n* = 294)	*p* Value *
**Characteristics**					
Age, years		20.0 (20.0–21.0)	20.0 (20.0–21.0)	20·0 (20.0–21.0)	0.737
	>50	1 (0.3%)	0 (0.0%)	1 (0.3%)	1.000
	≤50	376 (99.7%)	83 (100.0%)	293 (99.7%)	
Sex					1.000
	Male	375 (99.5%)	83 (100.0%)	291 (99.3%)	
	Female	2 (0.5%)	0 (0.0%)	2 (0.7%)	
**Symptoms**					
Fever		377 (100.0%)	83 (100.0%)	294 (100.0%)	NA
Maximum temperature, °C		38.6 (38.3–39.1)	38.6 (38.4–39.1)	38.6 (38.3–39.0)	0.669
	38–39	282 (74.8%)	61 (73.5%)	221 (75.2%)	0.775
	>39	95 (25.2%)	22 (26.5%)	73 (24.8%)	
Dyspnea		6 (1.6%)	4 (4.8%)	2 (0.7%)	0.023
Cough		377 (100.0%)	83 (100.0%)	294 (100.0%)	NA
Sputum		287 (76.1%)	63 (75.9%)	224 (76.2%)	1.000
Rhinorrhea		200 (53.1%)	39 (47.0%)	161 (54.8%)	0.216
Sore throat		275 (73.0%)	50 (60.2%)	225 (76.5%)	0.005
Headache		202 (53.6%)	43 (51.8%)	159 (54.1%)	0.803
Nausea		69 (18.3%)	18 (21.7%)	51 (17.3%)	0.421
Vomiting		23 (6.1%)	9 (10.8%)	14 (4.8%)	0.064
Diarrhea		22 (5.8%)	5 (6.0%)	17 (5.8%)	1.000

Data are median (IQR) or *n* (%). NA = not available. * Difference between pneumonia and non-pneumonia groups.

**Table 2:** Diagnostic Performance of DL algorithm and ED physicians (pneumonia vs. non-pneumonia).

	AUROC (95% CI)	*p* Value	Sensitivity (95% CI)	*p* Value	Specificity (95% CI)	*p* Value	Reading Time (min)
DL algorithm	0.861 (0.823–0.894)	NA	0.583 * (0.471–0.690)	NA	0.944 * (0.912–0.967)	NA	13
Session 1 (ED physicians only)							
Observer 1	0.788 (0.743–0.827)	0.019 ^a^	0.595 (0.483–0.701)	1.000 ^a^	0.690 (0.634–0.741)	<0.0001 ^a^	156
Observer 2	0.814 (0.771–0.851)	0.132 ^a^	0.500 (0.389–0.611)	0.119 ^a^	0.974 (0.949–0.989)	0.093 ^a^	160
Observer 3	0.808 (0.766–0.846)	0.043 ^a^	0.500 (0.389–0.611)	0.065 ^a^	0.997 (0.982–1.000)	0.0001 ^a^	179
Group	0.788 (0.763–0.811)	0.017 ^a^	0.532 (0.468–0.595)	0.053 ^a^	0.887 (0.864–0.907)	<0.0001 ^a^	165
Session 2 (ED physicians with DL algorithm assistance)							
Observer 1	0.838 (0.798–0.874)	0.111 ^b^	0.655 (0.543–0.755)	0.302 ^b^	0.954 (0.924–0.975)	<0.0001 ^b^	97
Observer 2	0.807 (0.765–0.846)	0.801 ^b^	0.560 (0.447–0.668)	0.227 ^b^	1.000 (0.988–1.000)	0.008 ^b^	87
Observer 3	0.806 (0.763–0.844)	0.913 ^b^	0.583 (0.471–0.690)	0.065 ^b^	0.990 (0.971–0.998)	0.625 ^b^	119
Group	0.816 (0.793–0.838)	0.068 ^b^	0.599 (0.536–0.660)	0.008 ^b^	0.981 (0.970–0.989)	<0.0001 ^b^	101

AUROC = the area under the receiver operating characteristics curve, DL = deep learning, ED = emergency department. * Sensitivity and specificity of DL algorithm were determined at high-sensitivity threshold. ^a^ Comparison of performance with DL algorithm. ^b^ Comparison of performance with session 1.

**Table 3:** Diagnostic Performance of DL algorithm and ED physicians (visible pneumonia on CR vs. non-pneumonia).

	AUROC (95% CI)	*p* Value	Sensitivity (95% CI)	*p* Value	Specificity (95% CI)	*p* Value
DL algorithm	0.940 (0.910–0.962)	NA	0.817 * (0.696–0.905)	NA	0·944 * (0·912–0·967)	NA
Session 1 (ED physicians only)						
Observer 1	0.856 (0.816–0.891)	0.003 ^a^	0.833 (0.715–0.917)	1.000 ^a^	0.690 (0.634–0.741)	<0.0001 ^a^
Observer 2	0.887 (0.850–0.918)	0.053 ^a^	0.700 (0.568–0·812)	0.119 ^a^	0.974 (0.949–0.989)	0.093 ^a^
Observer 3	0.920 (0.887–0.946)	0.455 ^a^	0.683 (0.550–0.797)	0.022 ^a^	0.997 (0.982–1.000)	0.0001 ^a^
Group	0.871 (0.849–0.890)	0.007 ^a^	0.739 (0.668–0.801)	0.034 ^a^	0.887 (0.864–0.907)	<0.0001 ^a^
Session 2 (ED physicians with DL algorithm assistance)						
Observer 1	0.936 (0.905–0.958)	0.007 ^b^	0.867 (0.754–0.941)	0.774 ^b^	0.954 (0.924–0.975)	<0.0001 ^b^
Observer 2	0.907 (0.873–0.935)	0.412 ^b^	0.783 (0.658–0.879)	0.227 ^b^	1.000 (0.988–1.000)	0.008 ^b^
Observer 3	0.907 (0.872–0.934)	0.609 ^b^	0.817 (0.696–0.905)	0.022 ^b^	0.990 (0.971–0.998)	0.625 ^b^
Group	0.916 (0.898–0.931)	0.002 ^b^	0.822 (0.758–0.875)	0.014 ^b^	0.981 (0.970–0.989)	<0.0001 ^b^

AUROC = the area under the receiver operating characteristics curve, CR = chest radiograph, DL = deep learning, ED = emergency department. * Sensitivity and specificity of DL algorithm were determined at high-sensitivity threshold. ^a^ Comparison of performance with DL algorithm. ^b^ Comparison of performance with session 1.

## Abbreviations

ARI	acute respiratory illness
FRI	febrile respiratory illness
ED	emergency department
CR	chest radiograph
DL	deep-learning
